# Protection of Neuroblastoma Neuro2A Cells from Hypoxia-Induced Apoptosis by Cyclic Phosphatidic Acid (cPA)

**DOI:** 10.1371/journal.pone.0051093

**Published:** 2012-12-12

**Authors:** Mari Gotoh, Katsura Sano-Maeda, Hiromu Murofushi, Kimiko Murakami-Murofushi

**Affiliations:** The Division of Life Sciences, Ochanomizu University, Tokyo, Japan; Universidade Federal do Rio de Janeiro, Brazil

## Abstract

Cyclic phosphatidic acid (cPA) is a naturally occurring phospholipid mediator with a unique cyclic phosphate ring at the *sn*-2 and *sn*-3 positions of its glycerol backbone. We have previously shown that cPA significantly suppresses ischemia-induced delayed neuronal death and the accumulation of glial fibrillary acidic protein in the CA1 region of the rat hippocampus. These results indicated that the systemic administration of cPA can protect hippocampal neurons against ischemia-induced delayed neuronal cell death. In the current study, we investigated the effects of cPA on neuronal cell death caused by hypoxia *in vitro* and the molecular mechanisms underlying these effects. We used cobalt chloride (CoCl_2_) to expose cells to hypoxic conditions *in vitro*. Treating mouse neuroblastoma (Neuro2A) cells with CoCl_2_ induced nuclear DNA condensation and phosphatidylserine exposure. However, adding cPA led to the suppression of CoCl_2_-induced apoptosis in a cPA dose-dependent manner and attenuated the increase in the Bax/Bcl-2 ratio caused by CoCl_2_. Quantitative PCR analysis showed that Neuro2A cells strongly express the LPA_1_, LPA_2_, and LPA_6_, which are G-protein coupled receptors that can be activated by cPA. To date, LPA_1_ and LPA_2_ have been reported to exhibit antiapoptotic activity. Therefore, to assess the roles of LPA_1_ and LPA_2_ on cPA-induced neuroprotective functions, Ki16425, a selective LPA_1_ and LPA_3_ antagonist, was adopted to know the LPA_1_ function and siRNA was used to knockdown the expression of LPA_2_. On the basis of our results, we propose that cPA-induced protection of Neuro2A cells from CoCl_2_-induced hypoxia damage is mediated via LPA_2_.

## Introduction

In 1992, cyclic phosphatidic acid (cPA) was originally isolated from the myxoamoebae of a true slime mold, *Physarum polycephalum* [1]. Later, the presence of cPA 16∶0 and 18∶1 has been found in mammalian fluids such as serum and brain tissue [2], [3]. And these are major forms of naturally occurring cPA species. Although the chemical formula of cPA is similar to that of lysophosphatidic acid (LPA), cPA has a unique structure consisting of a cyclic phosphate ring at the *sn*-2 and *sn*-3 positions of its glycerol backbone [1]. These features provide cPA with biological functions that are distinct from or oppose the functions of LPA. For example, LPA stimulates cell proliferation, cancer cell invasion, and generates pain, whereas cPA inhibits these activities [4]–[10].

Previous *in vitro* studies have reported that cPA 16∶0, 18∶1 and LPA 18∶1 elicit neurotrophin-like actions in embryonic hippocampal neurons [11]. We have examined the effects of cPA 18∶1 on ischemia-induced delayed neuronal death in the hippocampal CA1 region and found that the systemic administration of cPA 18∶1 has *in vivo* neuroprotective effects [12]. However, the mechanisms underlying these effects of cPA 18∶1 on hypoxic-ischemic brain injury have not yet been completely understood. Here, we aimed to investigate the effects of cPA 18∶1 on hypoxia-induced apoptosis and the molecular mechanism underlying these effects.

To induce hypoxic/ischemic conditions *in vitro*, we induced apoptosis with cobalt chloride (CoCl_2_), which is used as a chemical hypoxia-inducing agent for several types of neural cells [13]–[16]. Then, we investigated the possible mechanisms of LPA receptor involvement. To date, cPA has been reported to stimulate several LPA G protein-coupled receptors (GPCRs) such as LPA_1–5_ [6], [17]. LPA_1_, LPA_2_, and GPR87 have been shown to exhibit antiapoptotic activity [18]. cPA activates the LPA_1–5_ at significantly higher EC_50_ concentrations than LPA [6], [17]. Although cPA is a weak agonist against the LPA_1, 3, 4, 5_, it has a stronger efficacy against the LPA_2_ (140% maximal efficacy compared with LPA) [6]. Each LPA receptor exhibits a different affinity and efficacy toward cPA and LPA. In addition, each LPA receptor has a individual signaling pathway and function. Thus, cells expressing several LPA receptors might undergo a different cellular phenomenon when stimulated by cPA or LPA.

In this study, using neuroblastoma Neuro2A cells, we examined the effects of the neuroprotective functions that are specific to cPA 18∶1 and LPA 18∶1 and involve LPA receptors and also investigated the molecular mechanisms underlying these effects. Our results show that although both lipid mediators exhibit neuroprotective functions for Neuro2A, the signal pathway initiated by cPA and LPA may potentially differ.

## Materials and Methods

### 1. Pharmacologic agents

cPA 18∶1 was chemically synthesized as previously described [19]. Bovine serum albumin (BSA; fraction V, fatty acid free) and 1-oleoyl-*sn*-lysophosphatidic acid (LPA 18∶1) were purchased from Sigma-Aldrich (St. Louis, MO). LPA and cPA were dissolved in PBS containing 0.1% (w/v) BSA. The inhibitor of the LPA_1, 3_, that is, Ki16425 (Cayman Chemicals, MI), was dissolved in DMSO and added to Neuro2A cells at a working concentration of 10 µM at 20 min prior to adding cPA or LPA.

### 2. Cell culture and treatment

Mouse Neuro2A cells were obtained from the Department of Biochemistry, Faculty of Medicine, the University of Tokyo. The cells were originally obtained from the American Type Culture Collection (ATCC cat. no. CCL131). The cells were cultured in Dulbecco's modified Eagle medium (DMEM; Nissui, Tokyo, Japan) supplemented with 10% fetal bovine serum (FBS; Biowest, FL), 0.2% NaHCO_3_, and 0.06% glutamine. Cells were grown at 37°C in a humidified incubator containing 5% CO_2_. For all the experiments, 1.0×10^6^ Neuro2A cells were seeded into a 100-mm dish and incubated for 4 hours with culture media containing 10% FBS. The cells were then subjected to serum starvation for 16 hours with serum-free DMEM.

### 3. Cell viability

To determine cell viability, cells were seeded (4×10^4^ cells/well) in a 96-well plate. Serum-starved Neuro2A cells were exposed to various concentrations of CoCl_2_ for 24 hours. The cells were then stained with 10 µM calcein-AM (Dojindo, Kumamoto, Japan) in the dark for 30 min at 37°C and washed with phosphate-buffered saline (PBS). The fluorescence intensity (em/ex, 485/530 nm) of each well was measured using a CytoFluor series 4000 fluorescence microplate reader (Applied Biosystems, Tokyo, Japan). Data were calculated as the percent cell viability compared to that of controls without CoCl_2_ treatment and have been presented as the mean and SE values for triplicate wells.

### 4. DAPI staining

Neuro2A cells were washed with PBS and fixed with 4% paraformaldehyde for 10 min at room temperature. Then, the nuclei were stained with 4′,6-diamidino-2-phenylindole dihydrochloride (DAPI; Wako Pure Chemical Industries, Ltd., Osaka, Japan) for 10 min at room temperature. The chromatin structures of the cells were observed under a fluorescence microscope equipped with a UV combination filter (Nikon Corp., Tokyo, Japan).

### 5. Measurement of reactive oxygen species generation

Intracellular reactive oxygen species (ROS) generation was measured using the fluorescent probe 5-(and-6)-chloromethyl-2′,7′-dichlorodihydrofluorescein diacetate, acetyl ester (CM-H_2_DCFDA; Molecular Probes, Inc., OR). Serum-starved Neuro2A cells in 96-well plates were exposed to 300 µM CoCl_2_ and stained with 10 µM CM-H_2_DCFDA in the dark for 30 min. Subsequently, the cells were washed with PBS, and the fluorescence intensity (em/ex, 485/530 nm) of each well was measured using the CytoFluor series 4000 microplate reader. The data were calculated as the relative ROS induction compared to that of controls (without CoCl_2_ treatment, time 0) and have been presented as the mean and SE values of triplicate wells.

### 6. Flow cytometric analysis

Serum-starved Neuro2A cells were exposed to 300 µM CoCl_2_ in the presence of 10 µM cPA or LPA. After 24 hours, the extent of apoptosis in Neuro2A cells was quantified by flow cytometric analysis by using the Cell Lab Quanta™ SC MPL (Beckman Coulter, CA). Cell staining was performed with FITC-conjugated Annexin V (Trevigen, MD) or DEVD-FMK (a caspase-3 inhibitor that irreversibly binds to activated caspase-3, BioVision, CA) in accordance with the manufacturer's instructions.

### 7. Cell adhesion assay

Serum-starved Neuro2A cells were exposed to 300 µM CoCl_2_ in the presence of 10 µM cPA or LPA. After 24 hours, the non-adherent cells were removed by washing 3 times with PBS. The number of adherent cells (cells/cm^2^) was determined using an inverted phase-contrast microscope.

### 8. RNA interference

Neuro2A cells were transfected with Accell SMARTpool siRNA specific for the mouse LPA_2_ or Accell non-targeting siRNA (Dharmacon, Inc., CO) with Accell siRNA delivery media, according to the manufacturer's protocol. The cells were used 3 d after transfection.

### 9. RNA isolation and real-time PCR

Total RNA was extracted from Neuro2A cells by using the Isogen reagent (Nippongene, Toyama, Japan). It was used as a template for subsequent cDNA synthesis with oligo dT primers by using the Omniscript RT Kit (Qiagen, CA). mRNA levels were quantified using an ABI 7300 real-time PCR machine (Foster City, CA) and SYBR *Premix Ex Taq* II (Takara Bio Inc., Siga, Japan). Gene-specific primer sets were used as previously reported [12], [20]. To quantify the knockdown levels of LPA_2_ mRNA, the following 2 primer pair sets were used: primer set I, 5′-GTTGAGGTCACTCCCACGTT-3′ (F) and 5′- CGTGCCTTTCCCTAAACCTT-3′ (R); and primer set II, 5′-AGGCTGGATATGGTCATTGC-3′ (F) and 5′- GCAGCTCCAGCAGAAATGTA-3′ (R). The data were analyzed using the delta Ct method. The expression level of each LPA receptor was normalized to β-actin expression as previously described [12], [20].

### 10. Western blot analysis

Neuro2A cells were collected and subjected to western blot analysis to detect Bax and Bcl-2 protein expression. Proteins were separated by SDS-PAGE by using a 15% polyacrylamide gel and then transferred to an Immobilon-P Transfer Membrane (Millipore). Using anti-Bax or anti-Bcl-2 antibodies (1∶1000 dilution, Cell Signaling Technology, Inc., MA) and horseradish peroxidase-conjugated anti-rabbit IgG (1∶10,000 dilution; Kirkegaard & Perry Laboratories Inc., MD), immunodetection was performed using an enhanced chemiluminescence (ECL) system (GE Healthcare UK Ltd, Amersham Place, Little Chalfont, England).

### 11. Statistical analysis

All the values have been reported in terms of mean ± SE values. The data were analyzed using one-way analysis of variance (ANOVA) and subsequently with Dunnett's test. A *P* value less than 0.05 was considered to be statistically significant.

## Results and Discussion

### 1. CoCl_2_-induced apoptosis in Neuro2A cells

**Figure 1:**
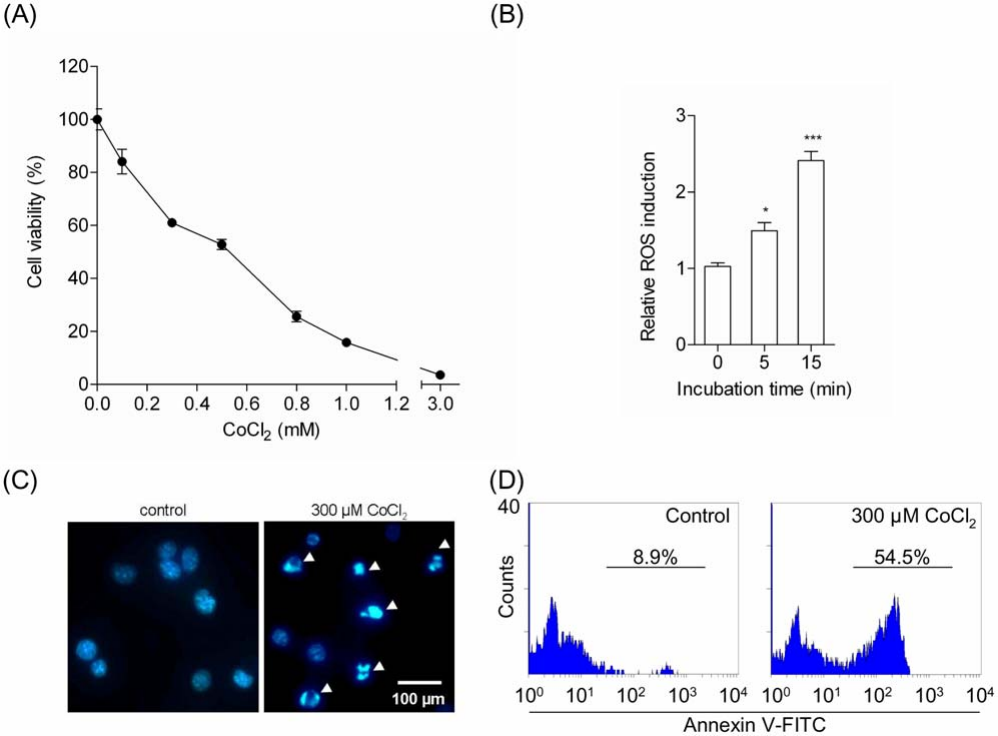
Treatment with CoCl_2_ induces apoptosis in Neuro2A cells. (A) Effects of CoCl_2_ on the viability of Neuro2A cells. Neuro2A cells were incubated with various concentrations of CoCl_2_ for 24 hours. Cell viability was estimated as described in the materials and methods section. The data represent the mean ± SE values from triplicate independent experiments. (B) Generation of reactive oxygen species (ROS) induced by CoCl_2_. Neuro2A cells were incubated with 300 µM CoCl_2_, and ROS generation was measured after 5 and 15 min. The data represent the mean ± SE values from triplicate independent experiments (*P<0.05, ***P<0.001 vs. the CoCl_2_-treated group). (C) Morphologic changes in the nuclei of CoCl_2_-treated Neuro2A cells. Neuro2A cells incubated in the absence (left) or presence (right) of 300 µM CoCl_2_ for 24 hours were fixed and stained with DAPI and examined by fluorescence microscopy. (D) Analysis of apoptosis-associated PS exposure on CoCl_2_-treated Neuro2A cells by using FITC-Annexin V. Neuro2A cells were treated with (right) or without (left) 300 µM CoCl_2_ for 24 hours and stained with FITC-Annexin V. The population of FITC-Annexin V-positive Neuro2A cells was quantified by flow cytometry.

Neuro2A cells were treated with various concentrations of CoCl_2_. After 24 hours, exposure of Neuro2A cells to CoCl_2_ significantly decreased cell viability in a CoCl_2_ dose-dependent manner (Fig. 1A). Exposure to 300 µM CoCl_2_ for 24 hours resulted in 61% viable cells compared to control cells (100%). The mode of cell death, necrosis, or apoptosis was determined by DAPI staining. After exposure to CoCl_2_, the cells displayed apoptotic morphology characterized by the condensation of chromatin, as shown in Fig. 1B. Moreover, to assess intracellular ROS generation, we measured the oxidation of CM-H_2_DCFDA [13]. CoCl_2_ treatment has been reported to significantly increase ROS levels within 1 h of incubation [21]. We also observed that treatment of Neuro2A cells with CoCl_2_ for 15 min induced oxidative stress by enhancing ROS levels (Fig. 1C). Our data show that exposure of Neuro2A cells to CoCl_2_ rapidly increased ROS levels and might initiate apoptosis signaling. Meanwhile it was revealed that Neuro2A did not generate superoxide by treatment of CoCl_2_ for 0–30 min (data not shown).

Flow cytometric analysis with FITC-Annexin V was used to analyze the rate of apoptosis induced by CoCl_2_ (Fig. 1D). Representative data show that exposure to 300 µM CoCl_2_ for 24 hours resulted in 54.5% FITC-Annexin V-positive Neuro2A cells in the entire cell population. On the other hand, no exposure to CoCl_2_ for 24 hours resulted in only 8.9% FITC-Annexin V-positive Neuro2A cells in the entire cell population. These results suggest that stimulation by 300 µM CoCl_2_ for 24 hours induced apoptosis in Neuro2A cells. Therefore, these conditions were used to induce apoptosis in Neuro2A cells in all subsequent experiments.

### 2. cPA protected Neuro2A cells against CoCl_2_-induced apoptosis

**Figure 2:**
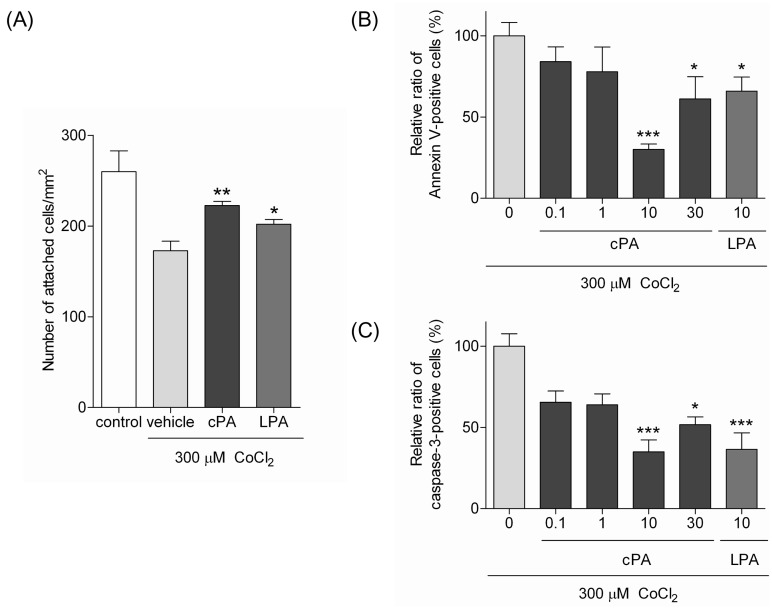
cPA protects against CoCl_2_-induced apoptosis in Neuro2A cells. (A) The effects of cPA and LPA on a number of adhesive Neuro2A cells treated with CoCl_2_. Neuro2A cells were incubated with 300 µM CoCl_2_ in the presence of 10 µM cPA or LPA for 24 hours, and the number of cells attached to the surface of dishes was determined (*P<0.05, **P<0.01 vs. the CoCl_2_-treated group). (B & C) Analysis of the percent of FITC-Annexin V- and FITC-DEVD-FMK-positive Neuro2A cells after CoCl_2_ treatment by flow cytometry. Neuro2A cells were incubated with 300 µM CoCl_2_ and various concentrations of either cPA or LPA for 24 hours. Cells were subsequently stained with either FITC-Annexin V (B) or FITC-DEVD-FMK, an activated caspase-3 inhibitor (C) and subjected to flow cytometric analysis. The data represent the mean ± SE values from triplicate independent experiments (*P<0.05, **P<0.01, ***P<0.001 vs. the CoCl_2_-treated group).

To examine the effects of cPA on CoCl_2_-induced apoptosis, Neuro2A cells were treated with CoCl_2_ in the presence or absence of cPA. Twenty-four hours later, the number of adherent cells (cells/cm^2^) was counted (Fig. 2A). At a concentration of 10 µM, cPA was observed to inhibit CoCl_2_-induced cell detachment. Although LPA is less potent than cPA, it also inhibited cell detachment. These results suggest that cPA and LPA could potentially attenuate CoCl_2_-induced Neuro2A cytotoxicity.

We then investigated the effects of cPA and LPA on CoCl_2_-induced apoptosis by measuring exposure of phosphatidylserine (PS) and activation of caspase-3. Exposure of PS on the surface of the cell membrane is related to the occurrence of early stages of apoptotic cell death and can be detected using Annexin V (PS-binding protein). Flow cytometric analysis with FITC-Annexin V showed that cPA-treatment significantly decreased the number of FITC-Annexin V-positive Neuro2A cells in a bell-shaped dose-dependent manner after exposure to CoCl_2_. At the most effective cPA-concentration (10 µM), the number of FITC-Annexin V-positive cells decreased to 30% of those in the vehicle control. LPA (10 µM) also exhibited neuroprotective effects on Neuro2A cells, as shown in Fig. 2B. However, the neuroprotective effects were not exhibited at lower concentrations (0.1 and 1 µM) of LPA (data not shown).

The cleavage of caspase-3 has been shown to be an important trigger for the execution of apoptosis [22], [23]. Treatment of Neuro2A cells with 300 µM CoCl_2_ significantly stimulated caspase-3 activity, which was attenuated by cPA in a bell-shaped dose-dependent manner, as shown in Fig. 2C. To confirm flow cytometric analysis, we performed western blot analysis to measure protein level of pro-caspase-3 using anti-caspase-3 antibody. It was revealed that level of pro-caspase-3 was significantly decreased by treatment of Neuro2A cells with 300 µM CoCl_2_ for 24 hours (data not shown), indicating pro-caspase-3 was activated. The decrease of pro-caspase-3 was attenuated by the addition of cPA 18∶1 or LPA 18∶1. These results are consistent with the results from flow cytometric analysis as shown in Fig. 2C.

In the case of measuring caspase-3 activity, 10 µM LPA but not lower concentration of LPA was observed to attenuate the caspase-3 activity. These results strongly indicate that cPA decreases the exposure of PS and caspase-3 activity and attenuates CoCl_2_-induced apoptosis.

### 3. Effects of CoCl_2_ and cPA on the expression of the Bcl-2 protein family in Neuro2A cells

**Figure 3:**
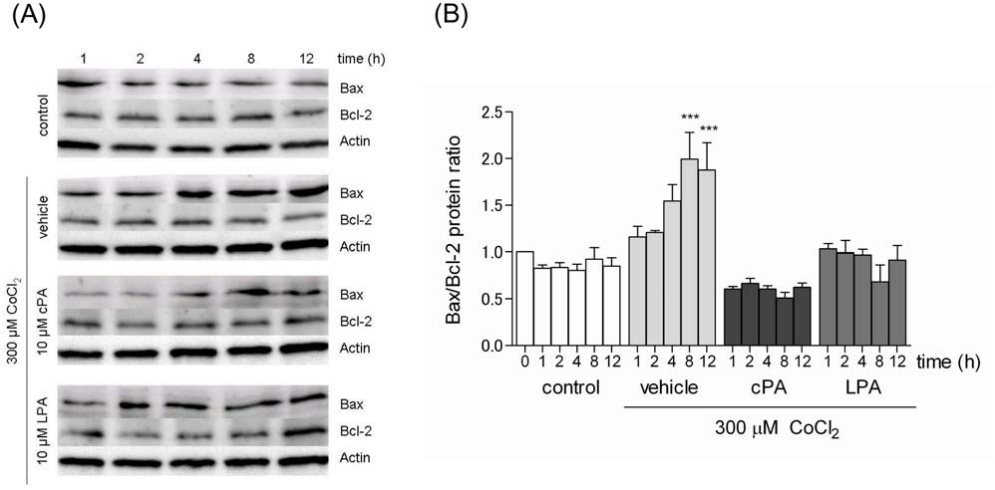
Effects of cPA and LPA on the expression of the Bcl-2 protein family. (A) Neuro2A cells were incubated with 300 µM CoCl_2_ in the presence of 10 µM cPA or LPA for the time indicated. Protein levels of Bax, Bcl-2, and β-actin were determined by western blot analysis. (B) The expression of Bax and Bcl-2 was determined using a densitometer, and the Bax/Bcl-2 ratio was calculated and normalized to cells without any treatment. The data represent the mean ± SE values from triplicate independent experiments (***P<0.001 vs. CoCl_2_-treated group).

The Bcl-2 family is an important regulator of various apoptotic pathways [22]. Because hypoxia induces an increase in the expression of proapoptotic Bcl-2 family members, such as Bax, and decrease in the expression of antiapoptotic Bcl-2 family members, such as Bcl-2, we examined the effects of CoCl_2_-induced apoptotic signaling on Bax and Bcl-2. Bax and Bcl-2 protein levels were determined by western blot analysis, and a time-course analysis of Bax and Bcl-2 protein expression ratios was performed (Fig. 3). The Bax/Bcl-2 ratio increased over time with CoCl_2_ treatment. However, in the presence of either cPA or LPA (10 µM), the ratio did not increase and remained at the ratio seen in control cells for up to 12 hours. Thus, cPA and LPA suppressed the CoCl_2_-induced increase in the Bax/Bcl-2 ratio and prevented CoCl_2_-induced apoptosis.

### 4. The antiapoptotic function of cPA is mediated by LPA_2_

**Figure 4:**
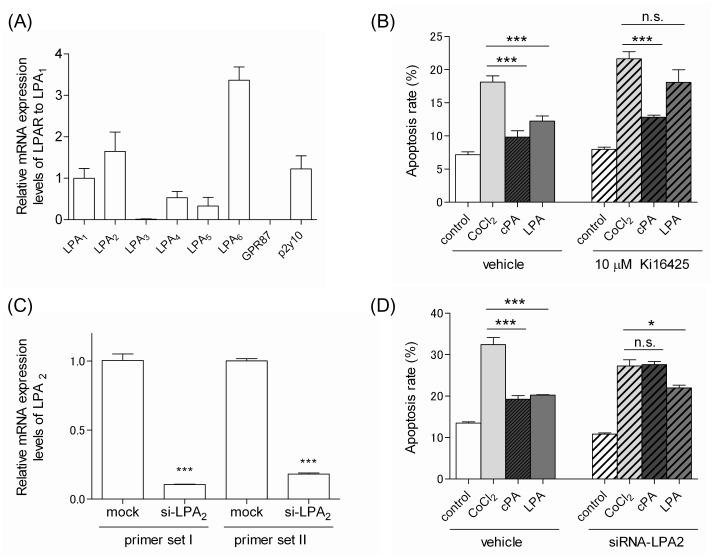
Effects of LPA receptors on the neuroprotective functions of cPA and LPA against CoCl_2_-induced apoptosis. (A) Expression of LPA receptors in Neuro2A cells. Total RNA was extracted from Neuro2A cells, and the expression level of each LPA receptor was determined by quantitative real-time PCR. The expression levels were normalized to those of LPA_1_ and expressed in terms of the mean ± SE values. (B) The effects of Ki16425 on the neuroprotective functions of cPA and LPA against CoCl_2_-induced apoptosis of Neuro2A cells. Neuro2A cells were pretreated with or without 10 µM Ki16425 for 20 min. Subsequently, the cells were incubated with 300 µM CoCl_2_ in the presence of 10 µM cPA or LPA for 24 hours. The cells were then stained with FITC-Annexin V and subjected to flow cytometric analysis. The data represent the mean ± SE values from triplicate independent experiments (***P<0.001 vs. the CoCl_2_-treated group). (C) Expression of LPA_2_ in Neuro2A cells. Neuro2A cells were transfected with siRNA against LPA_2_ or non-target siRNA. Total RNA was extracted from each transfected Neuro2A cell, and the expression level of each LPA receptor was determined by quantitative real-time PCR. The expression levels of LPA_2_ was normalized to those of Neuro2A cells transfected with non-target siRNA. The resulting data represent the mean ± SE values (***P<0.001 vs. the mock group). (D) The effects of LPA_2_ knockdown on the neuroprotective effects of cPA and LPA against CoCl_2_-induced apoptosis of Neuro2A cells. Neuro2A cells transfected with either siRNA against LPA_2_ or non-target siRNA were incubated with 300 µM CoCl_2_ in the presence of 10 µM cPA or LPA for 24 hours. Cells stained with FITC-Annexin V were subjected to flow cytometric analysis. The data represent the mean ± SE values from triplicate independent experiments (*P<0.05, ***P<0.001 vs. the CoCl_2_-treated group; n.s., not significant).

To gain insight into the molecular mechanism underlying the protective effects of cPA on CoCl_2_-induced apoptosis in Neuro2A cells, we focused on the signaling pathways initiated by LPA receptors. cPA has been shown to activate LPA_1–5_ [6], [17]. Recently, we examined whether cPA could activate LPA_6_ by measuring the LPA_6_-mediated neurite retraction of B103 cells as described previously [24], [25]. We observed that cPA could activate LPA_6_, although its efficacy and affinity were less potent than those of LPA (unpublished data). Quantitative PCR analysis showed that Neuro2A cells highly expressed LPA_1, 2, 6_ and p2y10 (Fig. 4A). Although LPA_1_, LPA_2_, and GPR87 have been speculated to have antiapoptotic activity [18], Neuro2A cells did not express GPR87. Therefore, we focused on the functions of the LPA_1_ and LPA_2_ in cPA-mediated antiapoptotic activity. In order to inhibit LPA_1_, we used Ki16425 (10 µM), which is an LPA receptor antagonist with selectivity for the LPA_1_ and LPA_3_ [26]. As shown in Fig. 4B, Ki16425 pretreatment attenuated LPA-mediated antiapoptotic activity for Neuro2A cells. However, the antiapoptotic activity of cPA was not affected by Ki16425. These results indicate that the antiapoptotic activity for Neuro2A of LPA may be mediated by LPA_1_, whereas that of cPA was not mediated by LPA_1_. We then used siRNA to knockdown LPA_2_ and assess the effects of reduced LPA_2_ expression on the antiapoptotic activity for Neuro2A cells of cPA and LPA. Compared to the mRNA expression level of LPA_2_ in non-targeting siRNA–transfected cells (control), the expression level in LPA_2_-targeted siRNA–transfected cells decreased by 14.4% (Fig. 4C). In addition, the antiapoptotic activity of cPA on CoCl_2_-induced Neuro2A cell apoptosis was largely abolished after knockdown of LPA_2_ (Fig. 4D). In the case of LPA, the effect of the suppression of its antiapoptotic activity on CoCl_2_-induced Neuro2A cell apoptosis was less apparent. Therefore, we propose that cPA-induced protection of Neuro2A cells from CoCl_2_-induced apoptosis might potentially be mediated by LPA_2_ and that LPA-induced protection of Neuro2A cells might be mediated mainly by LPA_1_ but not LPA_2_.

The LPA_1_ and LPA_2_ differ in their dose response to cPA and LPA. LPA_1_ can be activated by lower concentrations of cPA and LPA than LPA_2_ [6]. This difference in dose response has been speculated to have important biological implications [27]. These receptors can cooperate and respond over a wide range of LPA concentrations. For example, LPA_1_ is activated by LPA with 100% efficacy (EC_50_, 130 nM) and cPA with 66% efficacy (EC_50_, 1.7 µM). LPA_2_ is activated by LPA with 100% efficacy (EC_50_, 3 nM) and cPA with 140% efficacy (EC_50_, 180 nM) [6]. We speculate that the affinity for the ligand, efficacy of the ligand, and expression level of receptors in individual cells are important factors for LPA receptor signaling. Most cells express multiple LPA receptors and work cooperatively. Therefore, determining the signal response of individual LPA receptors is not easy. LPA_1_ and LPA_2_ are coexpressed in Neuro2A cells. The neuroprotective functions for Neuro2A of cPA could possibly be mediated by LPA_2_. Furthermore, the neuroprotective function of LPA might be mediated mainly by LPA_1_ but not LPA_2_ in Neuro2A cells. On the basis of these results, we suggest that 10 µM LPA could activate LPA_1_ but not LPA_2_. An LPA concentration of 10 µM is too high to activate LPA_2_. At high concentrations, LPA_2_ can undergo endocytic downregulation and degradation. On the other hand, 10 µM cPA activates only LPA_2_. The affinity of LPA_2_ for cPA is lower than that for LPA, which may prevent the receptors from undergoing endocytic downregulation on exposure to 10 µM of cPA. This may also be because the efficacy of cPA against LPA_2_ is higher than that of LPA. More remarkable effects were observed when the LPA_2_ was activated by cPA than when it was activated by LPA. Moreover, because LPA_2_ has a higher affinity for cPA than LPA_1_ does [6], cPA might prefer LPA_2_ over LPA_1_. Here, we have shown that cPA and LPA exhibit antiapoptotic activity for Neuro2A cells but their originating signaling pathways differ. We speculate that the receptors have differences in efficacy and affinity for cPA and LPA. Although many questions remain to be answered, the current study shows that lipid mediators that exhibit antiapoptotic activity and have overlapping receptors function via different receptors in apoptosis. We hypothesize that these differences in receptor preference and the efficacy of cPA and LPA can explain why cPA exhibits biological functions distinct from those of LPA.

We propose that cPA could be a promising therapeutic agent to treat ischemic brain injury. In order to develop novel compounds with medicinal properties, we have synthesized stable derivatives of cPA [6], [10], [28]. Moreover, we have also shown that each enantiomer of several cPA derivatives exhibits the same efficiency toward autotaxin inhibition [29]–[32]. On the basis of these findings, we are currently investigating effective therapeutic compounds to treat disorders such as cancer and neurodegeneration.

## Conclusion

CoCl_2_ treatment induced apoptosis in Neuro2A cells, involving an increase in ROS generation and the Bax/Bcl-2 ratio. cPA increased the cell survival rate, while decreasing the number of FITC-Annexin V- and FITC-DEVD-FMK-positive cells among CoCl_2_-treated Neuro2A cells in a bell-shaped dose-dependent manner. The protective effect of cPA on CoCl_2_-induced apoptosis in Neuro2A cells was not inhibited by the LPA_1_ and LPA_3_ antagonist Ki16425. However, the effect of LPA was inhibited by Ki16425. Knockdown of LPA_2_ in Neuro2A cells largely affected the neuroprotective response of cPA but not that of LPA. Therefore, we propose that the molecular mechanism underlying the protective effect of cPA might differ from that for LPA. In addition, we suggest that cPA-induced protection of Neuro2A cells from CoCl_2_-induced hypoxia damage might be mediated via LPA_2_.
